# An evolutionary case for plant rarity: *Eucalyptus* as a model system

**DOI:** 10.1002/ece3.11440

**Published:** 2024-06-06

**Authors:** Alivia G. Nytko, John K. Senior, Rachel C. Wooliver, Julianne O'Reilly‐Wapstra, Jennifer A. Schweitzer, Joseph K. Bailey

**Affiliations:** ^1^ Ecology and Evolutionary Biology University of Tennessee Knoxville Tennessee USA; ^2^ Biological Sciences, School of Natural Sciences University of Tasmania Hobart Tasmania Australia; ^3^ Biosystems Engineering & Soil Science University of Tennessee Knoxville Tennessee USA

**Keywords:** Blomberg's K, eucalyptus, performance traits, phylogeny, rare species, rarity

## Abstract

Species rarity is a common phenomenon across global ecosystems that is becoming increasingly more common under climate change. Although species rarity is often considered to be a stochastic response to environmental and ecological constraints, we examined the hypothesis that plant rarity is a consequence of natural selection acting on performance traits that affect a species range size, habitat specificity, and population aggregation; three primary descriptors of rarity. Using a common garden of 25 species of Tasmanian *Eucalyptus*, we find that the rarest species have 70% lower biomass than common species. Although rare species demonstrate lower biomass, rare species allocated proportionally more biomass aboveground than common species. There is also a negative phylogenetic autocorrelation underlying the biomass of rare and common species, indicating that traits associated with rarity have diverged within subgenera as a result of environmental factors to reach different associated optima. In support of our hypothesis, we found significant positive relationships between species biomass, range size and habitat specificity, but not population aggregation. These results demonstrate repeated convergent evolution of the trait‐based determinants of rarity across the phylogeny in Tasmanian eucalypts. Furthermore, the phylogenetically driven patterns in biomass and biomass allocation seen in rare species may be representative of a larger plant strategy, not yet considered, but offering a mechanism as to how rare species continue to persist despite inherent constraints of small, specialized ranges and populations. These results suggest that if rarity can evolve and is related to plant traits such as biomass, rather than a random outcome of environmental constraints, we may need to revise conservation efforts in these and other rare species to reconsider the abiotic and biotic factors that underlie the distributions of rare plant species.

## Graphical abstract

Although species rarity is often considered to be a stochastic response to environmental and ecological constraints, we examined the hypothesis that plant rarity is a consequence of natural selection acting on performance traits that affect a species range size, habitat specificity, and population aggregation; three primary descriptors of rarity. Using a common garden of 25 species of Tasmanian *Eucalyptus*, we find a negative phylogenetic autocorrelation underlying the significantly lower biomass of rare versus common species, indicating that traits associated with rarity have diverged within subgenera as a result of environmental factors to reach different associated optima. These results demonstrate repeated convergent evolution of the trait‐based determinants of rarity across the phylogeny in Tasmanian eucalypts and suggest that we may need to revise conservation efforts to reconsider the abiotic and biotic factors that underlie the distributions of rare plant species.

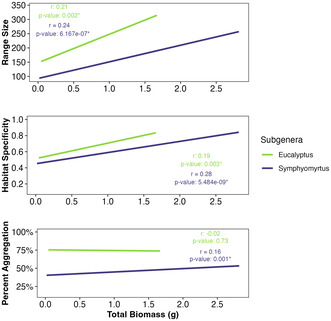

## 1 INTRODUCTION

Understanding the causes and consequences of global biodiversity loss is fundamental to the field of ecology. Under current projections, climate change and human disturbance is expected to disproportionately affect historically rare species (Enquist et al., 2019). Climate‐driven loss of rare species is important as nearly 37% of plant species are considered exceedingly rare on the landscape and have high conservation value, especially those in stable climates (Enquist et al., 2019). Rarity is typically characterized along three primary axes including range size, habitat specificity, and population abundance (Rabinowitz, 1981). These parameters have been used globally to conduct experiments, organize databases, and identify populations and species of conservation interest for decades (Choe et al., 2019; Espeland & Emam, 2011; Levine et al., 2010; Vincent et al., 2020), but see (Enquist et al., 2019). Although this three‐dimensional framework provides a convenient classification of different types of rarity, rare species can have unique traits relative to common species that are not typically considered in rare species management and conservation. For example, rare species often demonstrate low biomass (Kempel et al., 2018; Vincent et al., 2020), low seed output (Boyd et al., 2022), small reproductive structures (Boyd et al., 2022), and limited genetic variation (Boyd et al., 2022). Furthermore, rare species contribute disproportionately to ecosystem services through functional trait diversity (Dee et al., 2019; Jain et al., 2014). Although many of the unique traits of rare species have been characterized, it is unknown if these traits are selected for under the conditions of rarity (Rabinowitz, 1981). In particular, if a suite of traits are repeatedly selected across the phylogeny of rare species, an evolutionary syndrome of rarity may exist and act upon species' range size, habitat specificity, and population sizes. Therefore, the traits that are related to whether a species is rare and how it is rare on the landscape, may also affect patterns of biodiversity and ecosystem function (Angert et al., 2011; Broenniman et al., 2005; Cron et al., 2009; Des Roches et al., 2018; Gorman et al., 2014; Quiroga & Souto, 2022; Ricklefs et al., 2008; Sfair et al., 2018). Overall, we know little about the evolutionary causes and consequences of rarity, such as: (1) whether there are specific performance traits associated with plant rarity that may determine if and how a plant species is rare; (2) if those traits are under selection or have evolved; (3) if those traits are related to the three primary axes of rarity; and (4) if there are potential ecosystem level consequences associated with being rare that are not currently being considered.

The effects of disturbance, invasion, and human/climate‐mediated habitat loss have led to a significant decline in the abundance of many species across global landscapes (Enquist et al., 2019). Additionally, past patterns of glaciation have also exerted constraints on the post‐glacial expansion of species, causing range restrictions and shifts in rarity (McKinnon et al., 2004). Understanding how rare plant species will respond to global change is paramount for effective conservation efforts. Identifying potential evolutionary controls underlying the determinants of rare species' range size, habitat specificity, and local population sizes is critical in this regard. While rarity has traditionally been attributed to a culmination of unfavorable and stochastic ecological processes, recent studies such as Boyd et al. (Boyd et al., 2022) suggest that species rarity may be driven by complex interactions between ecological (climate, habitat loss, biotic interactions, etc.) and evolutionary (phylogenetic isolation, genetic variation, etc.) factors across spatial and temporal scales. Furthermore, performance traits, many of which are under selection, are closely linked to species rarity and correlated with differences in productivity, reproductive, and survival outcomes between rare and common species (Boyd et al., 2022). Specifically, plant biomass and patterns of biomass allocation, two important plant performance traits, play a central role in shaping structural and reproductive traits (Kleyer et al., 2019). These traits include stem length/mass, canopy height, root length, seed dynamics, and reproductive output, which in turn influence species' fitness and geographic distributions (Younginger et al., 2017). Furthermore, many of these plant traits such as wood density (Swenson & Enquist, 2007), growth form, maximum height, seed mass (Chazdon et al., 2003; Kraft & Ackerly, 2010), and water‐use traits (Ávila‐Lovera et al., 2023) exhibit strong phylogenetic conservatism and are thus subject to strong evolutionary selection. Consequently, understanding the evolutionary processes acting on trait‐based shifts in ecological function and geographic distribution allows for predictability in the adaptation, survival, and future persistence of the associated species. This is increasingly important for rare species that contribute disproportionately to ecosystem services but face increased threats under anthropogenic change. For example, if plant traits associated with rarity, such as biomass, are found to be genetically controlled and under selection, global decreases in plant biomass associated with progressive rarity may lead to major shifts in carbon sequestration on the landscape (Jain et al., 2014; Stephenson et al., 2014). Through the integration of phylogenetic and trait‐based concepts, we can better understand and address: why species are rare, which species may become rare, and how rare species may respond to climate.

Twenty‐nine native species of Tasmanian *Eucalyptus*, from two major subgenera, vary in rarity and provide a model study system to identify the genetic basis of plant traits that may be associated with rarity and subsequent ecosystem‐level consequences (Rabinowitz, 1981; Senior et al., 2013; Wooliver et al., 2017). In this study we hypothesized that: (1) Progressively rare species have lower above‐ and belowground biomass than common species. Specifically, we predicted that the rarest species (level 1) will have the lowest biomass, rare species that are not equally rare across all three axes of rarity (level 2–7) will have variably low biomass, and common species will have the highest biomass. (2) Seedling biomass has an underlying phylogenetic signal and is thus under genetic control. (3) Differences in range size, habitat specificity, and population aggregation are related to the biomass of rare and common species. Consequently, biomass will be positively correlated with the three axes of rarity, such that species with larger biomass will have larger range sizes, less specific habitats, and larger population sizes than species with smaller biomass. Understanding the genes to ecosystem consequences of species rarity is a new frontier that is critical to understanding the spatial and functional responses of rare plant species to climate change, as well as further predicting future ecosystem function across the landscape. If the traits of rare species are under selection and directly related to the three axes of rarity, the selection acting on these traits may determine if, how, and which species will become rare on the landscape.

## 2 METHODS

Twenty‐nine species of *Eucalyptus* are native to the island state of Tasmania, Australia. Species of Tasmanian *Eucalyptus* belong to two subgenera: *Symphyomyrtus* containing 17 Tasmanian species, and *Eucalyptus*, containing 12 Tasmanian species (Grattapaglia et al., 2012). Although many common eucalypt species, such as *Eucalyptus globulus*, are often characterized by their native prevalence and use in widespread plantations, many other, lesser‐known species provide valuable ecosystem services, such as increased soil fertility and carbon sequestration (Davies et al., 2019; McIntosh & Moroni, 2016), across the Tasmanian landscape. Tasmanian *Eucalyptus* provides a model study system to investigate the drivers of trait expression and geographic occurrence dynamics due to the wide variation in life form, productivity, and rarity expressed among native Tasmanian eucalypt species. Furthermore, the ranges, habitat preferences, and population dynamics of the Tasmanian eucalypt species have been extensively documented (Williams & Potts, 1996) and a comprehensive phylogeny has recently been resolved (Wooliver et al., 2017). The combination of ecological data and phylogenetic tools allows for powerful inference regarding eco‐evolutionary dynamics under climate change. For example, Senior et al. (Senior et al., 2013) demonstrated empirical support linking the response of eucalypts to climate‐induced increases in atmospheric CO_2_ and soil nitrogen (N) to phylogenetic structure. Although many studies have examined the role of phylogenetics in eucalypt nutritional acquisition and hybridization (Pfeilsticker et al., 2022; Wooliver et al., 2017), phylogeny has not yet been used to understand the selection for plant traits and distributions associated with rarity. Our study bridges this gap to empirically test if the above‐ and belowground biomass of Tasmanian eucalypt species is under genetic control and determine the importance of this process in shaping the rarity of species on the landscape.

Rabinowitz's framework of rarity provides an easy‐to‐follow and widely used system to differentiate between different degrees of rarity in order to best investigate the causes and consequences of rarity. The generality of this system is profound as it is not constrained by specific taxa or geographic location (Rabinowitz, 1981). Using Rabinowitz's three descriptors of rarity, we created a rank order continuum of rarity ranging from excessively rare (level 1) to least rare (level 7) and common (Figure S1 & Table S1). Specifically, species with small ranges, specific habitats, and small local population sizes were considered to be the most rare (level 1), while species with large ranges, ubiquitous habitats, and large local populations were considered to be common (Figure S1). To examine the relationship between above‐ and belowground biomass, rarity, and patterns of geographic occurrence, we categorized 25 (of the 29) species of native Tasmanian eucalypts into these seven different rarity levels; the remaining four Tasmanian eucalypts were omitted due to seed unavailability and mislabeling. Species range sizes were created in accordance with Williams & Potts (Williams & Potts, 1996) by connecting occupied 10 km × 10 km grid cells from the outer marginal extremes of a core distribution to outliers and interpolating the number of cells within the resulting envelope for each species. Similarly, the aggregation of each species was calculated as the average % occurrence within each grid cell of the species range. Population aggregation refers to the spatial and distribution dynamics of individuals in a population, whereas local abundance refers to the number of individuals in a population. Despite this distinction, both metrics are highly correlated and provide insight into population‐level trends in rarity (Flügge et al., 2012). Consequently, we utilized population aggregation as a proxy for local abundance, as suggested by Flügge et al. (Flügge et al., 2012). For example, a species that has a range size of four 10 km × 10 km grid cells and only occurs within 36% of those cells has a range of 400 km^2^ and an aggregation of 36%. Additionally, we calculated habitat specificity for each species as the proportion of bioregions inhabited in Tasmania (Kitchener & Harris, 2013). To measure the above‐ and belowground biomass of these eucalypt species, we established a full factorial common garden experiment consisting of monocultures and two species mixtures of different species under varying levels of CO_2_ and nitrogen (N) fertilization using seeds from each species obtained from one of six maternal trees among a single population (Figure S2). Consequently, the biotic effects of plant–plant interactions may potentially work in tandem with species rarity to shape biomass outcomes in two‐species mixture treatments. Additionally, while the use of single populations may limit generalizability across broader ranges of widespread species, the inclusion of multiple maternal trees within populations enhances the representation of genetic diversity and robustness of the experimental design. CO_2_ and N treatments did not significantly interact with species rarity in preliminary linear mixed models (Table S2). Therefore, the final statical models did not incorporate the effects of CO_2_ addition and N enrichment, as they are unrelated to the hypotheses proposed and are not the focus of the study. Findings regarding the CO_2_ and N enrichment treatments are presented and discussed in Senior et al. (Senior et al., 2013). After approximately five months of growth, seedlings were harvested and the aboveground and belowground biomass was separated, dried, and weighed (g). Above‐and belowground biomass was summed to determine total biomass. Tasmanian *Eucalyptus* seedling biomass has a significant positive correlation to mean adult height (*r* = .28, *p*‐value = 2.211e‐14); however, we recognize that seedling biomass may not fully represent temporal patterns in productivity (i.e., small seedling biomass could also be representative of slow small growth rates, rather than small adult biomass). See details of this experiment from Senior et al. (Senior et al., 2013); data from this paper were recategorized through the addition of rarity levels and reanalyzed using phylogenetic comparative methods to address hypotheses outlined above.

All statistical analyses were performed using R Statistical Software (version 4.2.1, R Core Team 2022). Aboveground, belowground, and total seedling biomass data were log transformed for normality. To test hypothesis 1 that rare species have lower above‐and belowground biomass than common congeners, we used phylogenetic generalized least squares (PGLS) models to determine the main effect of species rarity on the above‐ and belowground biomass of Tasmanian eucalyptus while accounting for underlying phylogenetic signal and variable nonindependence (“pgls” function in “caper” package, R). PGLS models perform linear regressions on comparative data (“comparative. data” function in “caper” package, R) that include both trait data and a phylogeny to account for phylogenetically based relationships between predictor and response variables. Consequently, model significance is likely driven by predictor variables/main effects, rather than phylogenetic structure and the nonindependence of traits due to a shared evolutionary history (Symonds & Blomberg, 2014). Differences in life form (tree, mallee, shrub) were also accounted for as a fixed effect in all PGLS models. Tukey HSD post‐hoc analyses were completed for all significant results (“ghlt” and “cld” functions in “multcomp” package, R). Additionally, we calculated the relative biomass allocation (i.e., above versus belowground biomass allocation) for each replicate by dividing the aboveground biomass by total biomass: (aboveground biomass/total biomass). Relative biomass allocation was then standardized around the mean: (((replicate aboveground biomass/replicate total biomass) – mean allocation proportion)/standard deviation of proportion). Negative standardized biomass allocation strengths represented increased allocation of resources to belowground biomass, while positive standardized biomass allocation strengths represented increased allocation of resources to aboveground biomass.

To test hypothesis 2 that seedling biomass has an underlying phylogenetic signal, we analyzed the phylogenetic signals of total, aboveground, and belowground biomass using Blomberg's K (“phylosig” function in “phytools” package, R). Specifically, a phylogenetic signal refers to the tendency for related species to have more similar or dissimilar trait values based on evolutionary history and genetic relationships. The Blomberg's *K* statistic estimates the strength and direction of phylogenetic signals set relative to an expected Brownian motion model of evolution (Adams, 2014; Blomberg et al., 2003). A Blomberg's *K* value equal to 1 is expected under a model of Brownian motion, while values of *K* > 1 indicate a positive signal in which traits are more similar among closely related species than expected under random drift, and values of *K* < 1 indicate negative phylogenetic autocorrelation in which traits are less similar than expected under random drift (Windernitz, 2016). To test hypothesis 3 that differences in range size, habitat specificity, and population aggregation are positively related to the biomass of species, we utilized PGLS models to examine the fixed effect of total biomass on range size, habitat specificity, and population aggregation separately, both within and across subgenera.

## 3 RESULTS

**FIGURE 1:**
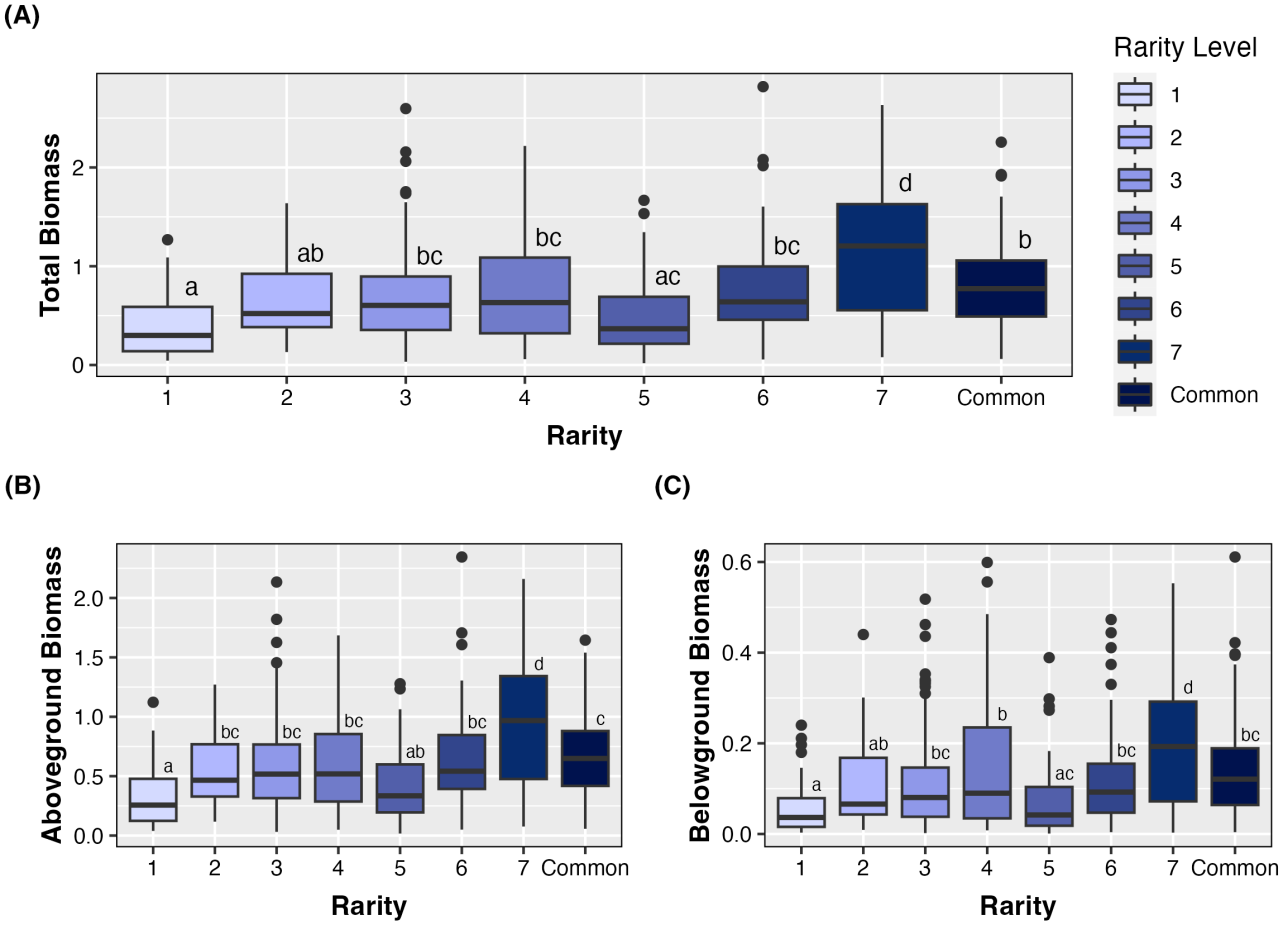
Aboveground, belowground, and total biomass responses to variation in rarity level. Comparative boxplots demonstrating the (A) total biomass, (B) aboveground biomass, and (C) belowground biomass of 25 Tasmanian *Eucalyptus* species categorized into seven forms of rarity.

**FIGURE 2:**
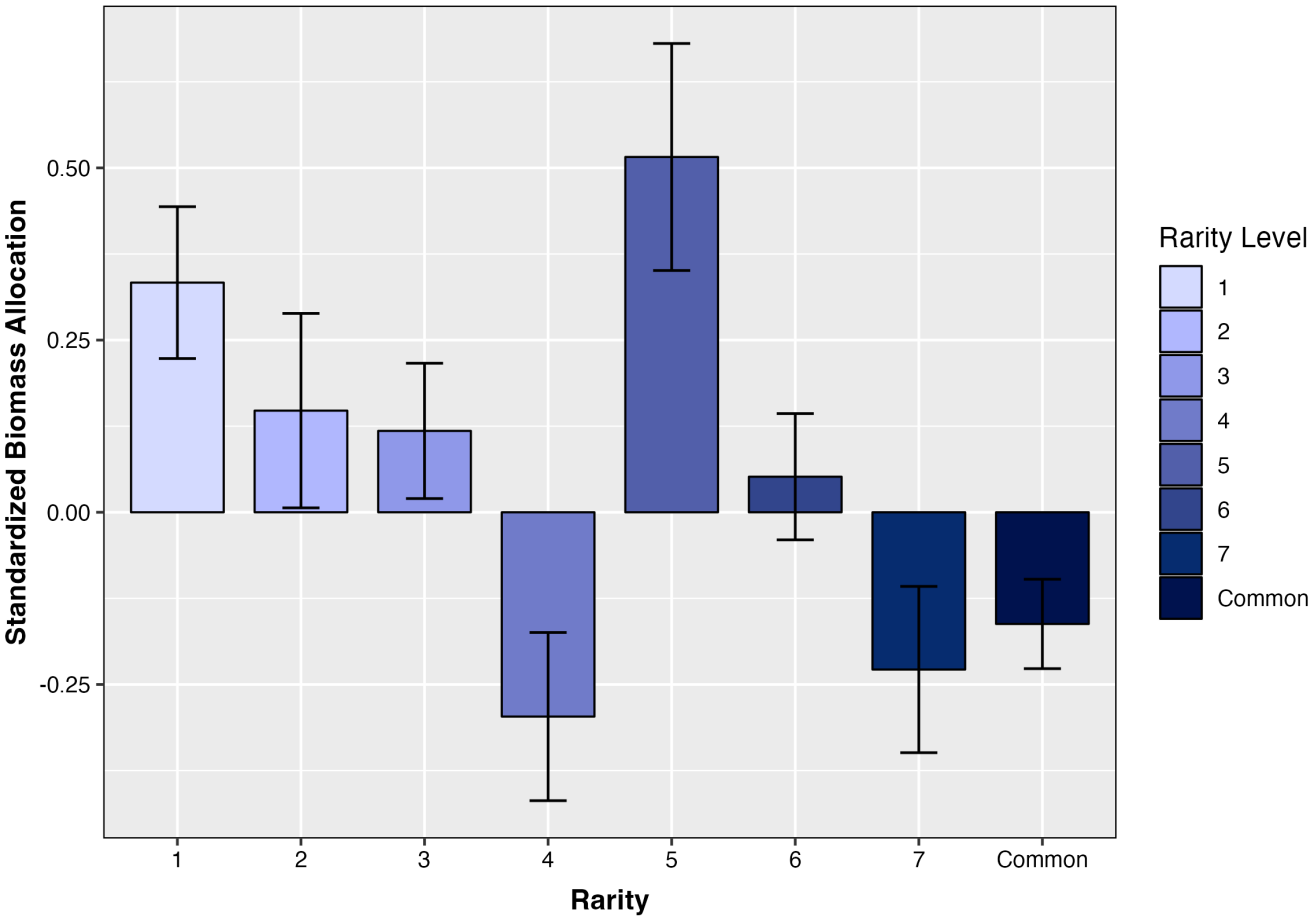
Standardized biomass allocation strength by rarity level. Positive standardized allocation strengths represent more resources proportionally allocated to aboveground biomass. Negative standardized allocation strengths represent more resources proportionally allocated to belowground biomass. Standardized allocation strengths near or at zero are indicative of equal partitioning of resources to above‐ and belowground biomass.

In support of hypothesis 1 that rare species have lower biomass than common species, the aboveground, belowground, and total biomass of Tasmanian *Eucalyptus* differed significantly depending upon rarity level (Figure 1, Table 1 & Table S3). Specifically, rare species seedlings were less productive than more common species when planted in a common garden. Average total seedling biomass varied from 0.384 g to 0.795 g from the rarest (level 1) to common species; a difference of 70%. These patterns were reflected in aboveground biomass which was 67% greater and belowground biomass which was 85% greater in common species compared to the rarest (level 1) species. While progressively less rare species demonstrated greater biomass, this trend was not linear and emphasizes the nuances of species varying in rarity (Table S3). For example, level 5 rare species demonstrated 27%–35% lower average total biomass than rarer species (levels 2–4), despite being characterized by small ranges, but wide habitats and large local population sizes. On the other hand, level 7 rare species demonstrated 37%–100% greater total biomass than all other rarity types and common species, despite maintaining narrow habitats. Interestingly, we also found that while rare species maintained lower above‐ and belowground biomass than common congeners, rare species proportionally allocated more biomass aboveground than belowground. In contrast, common species proportionally allocated more resources to belowground biomass. Specifically, the rarest species (level 1) had an average, positive standardized allocation strength of 0.333, whereas common species had an average, negative standardized allocation strength of −0.162 (Figure 2). Furthermore, differences in above‐ and belowground biomass allocation were based on species range size. Species characterized by small ranges allocated more biomass aboveground, while species characterized by large ranges allocated more biomass belowground, regardless of rarity level. Differences in life form (tree, mallee, shrub) did not significantly affect total biomass, aboveground biomass, or belowground biomass (Table 1).

**TABLE 1:** Results of PGLS models, examining the effect of ordinal rarity level and life form (tree, mallee, shrub) on aboveground, belowground, and total biomass.

Response	Covariate	df	*F*‐statistic	*p*‐Value
Total biomass	Rarity	7	3.034	.034*
	Life form	2	0.142	.869
Aboveground biomass	Rarity	7	3.149	.030*
	Life form	2	0.170	.846
Belowground biomass	Rarity	7	2.762	.047*
	Life form	2	0.079	.925

*Note*: Statistical significance (*) is denoted at an alpha level of 0.05.

**FIGURE 3:**
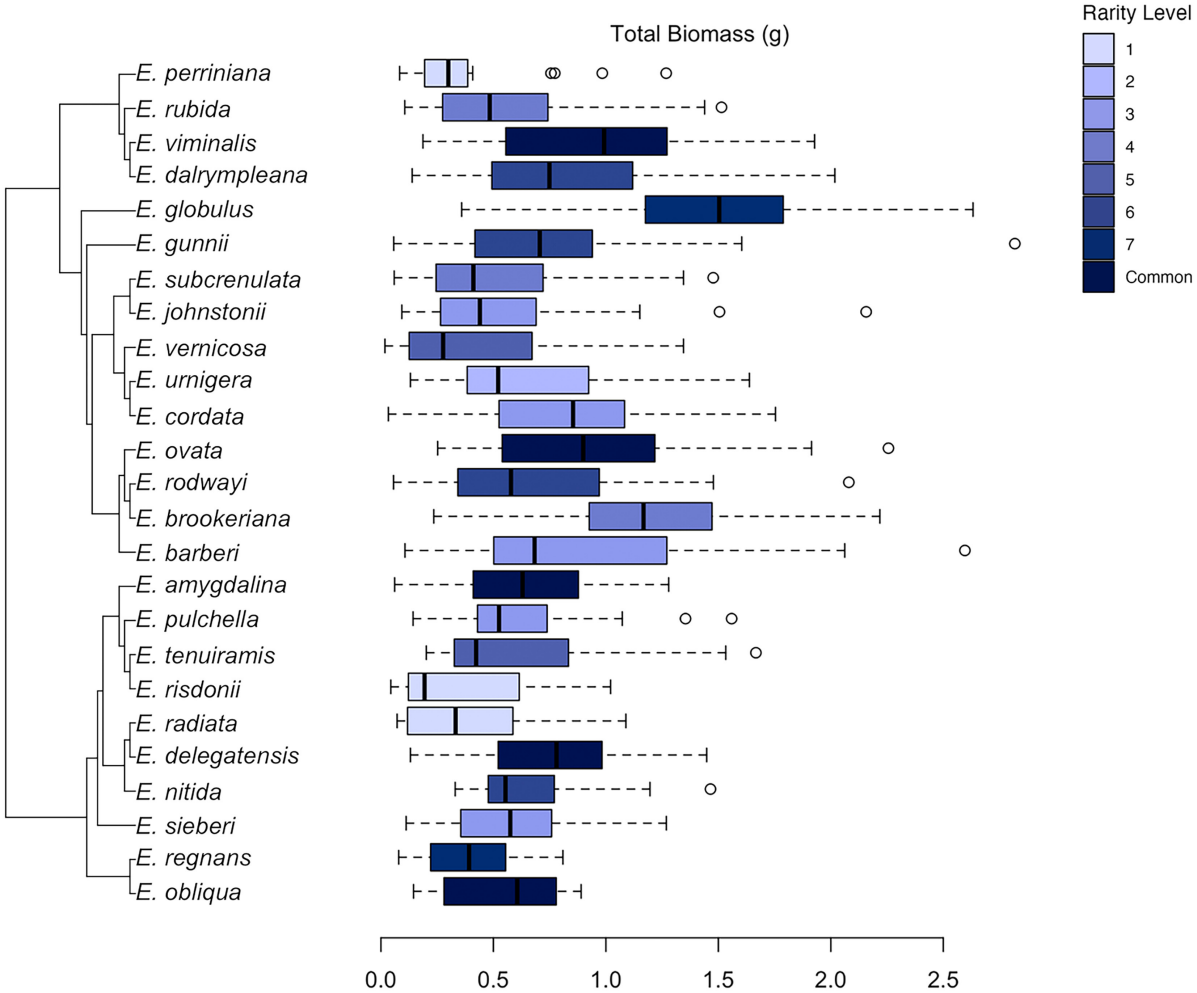
The biomass of Tasmanian eucalypts demonstrate an underlying negative phylogenetic autocorrelation. A negative phylogenetic autocorrelation or dissimilar signal underlying biomass production is seen underlying closely related species on the Tasmanian *Eucalyptus* phylogeny.

We also found support for hypothesis 2 that seedling biomass has an underlying phylogenetic signal. Both subgenera of Tasmanian *Eucalyptus* displayed strong negative phylogenetic autocorrelations for plant biomass, demonstrating the evolution of performance traits across the phylogeny (Figure 3 & Table 2). Seen in above‐ and total biomass, the absence of a positive phylogenetic signal, and presence of a significant dissimilar signal, suggests that plant biomass has diverged within subgenera to employ different evolutionary strategies. The variation in biomass of closely related sister taxa is exemplified by a 50% difference between *E. rodwayi* and *E. brookeriana* within subgenus *Symphomyrtus* and a 66% difference between *E. radiata* and *E. delegatensis* within subgenus *Eucalyptus*. On a larger scale, this negative phylogenetic autocorrelation can be seen between species belonging to the subgenus *Symphomyrtus* containing on average 37% more biomass than species belonging to the subgenus *Eucalyptus*.

**TABLE 2:** Calculated Blomberg's *K* values for total biomass, aboveground biomass, and belowground biomass.

Variable	*K*	*p*‐Value
Total biomass	0.018	.023*
Aboveground biomass	0.018	.018*
Belowground biomass	0.016	.069

*Note*: Blomberg's *K* values equal to 1 represent Brownian motion, while values of *K* > 1 represent positive phylogenetic signals indicative of highly similar trait values among closely related species, and values of *K* < 1 represent negative phylogenetic autocorrelations indicative of highly dissimilar trait values among closely related species. Statistical significance (*) is denoted at an alpha level of 0.05.

**FIGURE 4:**
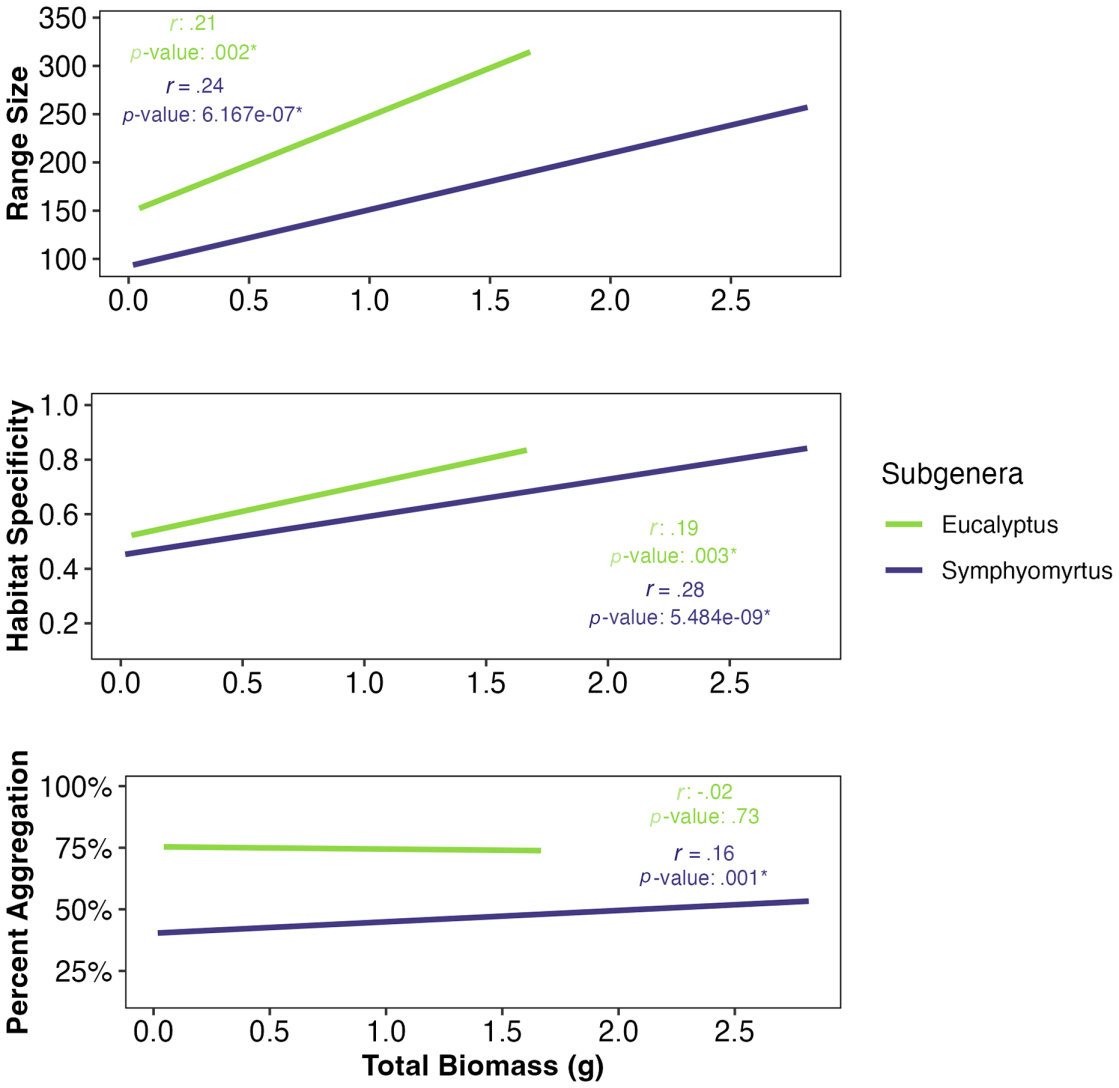
Range size, habitat specificity, and population aggregation responses to total biomass by subgenus of Tasmanian *Eucalyptus*. Relationships between range size and habitat specificity by total biomass and subgenera demonstrate significant divergence in biomass within subgenus to create a convergent pattern among subgenera.

Further supporting hypothesis 3 that rarity may be a consequence of evolutionary processes acting on plant performance plant traits, range size and habitat specificity also varied significantly in response to total biomass (Figure 4, Table 3 & Table S4). Both subgenera of Tasmanian *Eucalyptus* exhibited a positive linear relationship between field range size and experimental seedling biomass. Similarly, in both clades, as seedling biomass increased, the correlation between the number of potential habitats occupied also increased. These results demonstrate convergent patterns across the phylogeny driven by divergent evolution in biomass within subgenera. Species with the average largest biomass had, on average, 193% larger ranges and inhabited 6 more habitats relative to species with the average smallest biomass. We found no significant relationship between population aggregation and total biomass.

**TABLE 3:** Results of PGLS models, examining the singular and interactive effects of total biomass and subgenera (*Eucalyptus* and *Symphyomyrtus*) on range size, habitat specificity, and population aggregation.

Response	Covariate	df	*F*‐statistic	*p*‐Value
Range size	Total biomass	1	10.343	.004*
	Subgenera	1	0.006	.940
	Total biomass × Subgenera	1	3.501	.075
Habitat specificity	Total biomass	1	9.126	.007*
	Subgenera	1	0.004	.948
	Total biomass × Subgenera	1	3.393	.080
Population aggregation	Total biomass	1	0.813	.378
	Subgenera	1	0.007	.934
	Total biomass × Subgenera	1	3.531	.074

*Note*: Statistical significance (*) is denoted at an alpha level of 0.05.

## 4 DISCUSSION

Rare plant species are small (Kempel et al., 2018; Vincent et al., 2020), allocate less resources to belowground biomass, less fecund (Boyd et al., 2022), functionally distinct (Dee et al., 2019; Jain et al., 2014), and are typically considered inferior competitors (Vincent et al., 2020) in comparison to common species. These characteristics are often attributed to ecological as well as anthropogenic factors such as land use change, habitat reduction, invasive encroachment, and shifts to relevant climate envelopes. While these global change factors are important drivers of rarity, much less attention has been given to the evolutionary drivers of trait variation in rare versus common species and what this means for rare species persistence. Our results show that (1) there are important plant performance traits related to aspects of rarity (i.e., biomass production and allocation); (2) these traits are under phylogenetic control; and (3) these performance traits demonstrate convergent evolution across the phylogeny. Recent work has hypothesized that the rarity and commonness of species is mechanistically driven by evolutionary processes shaping distributional patterns of species abundance (Callaghan et al., 2023). Specifically, nonrandom, trait‐based processes may shape species' abundance and therefore rarity (Cornwell & Ackerly, 2010). Our work provides quantitative support for this hypothesis using Tasmanian eucalypts to demonstrate the role of performance traits, under divergent selection, in shaping patterns of geographic occurrence in rare versus common species. Although the 25 species of Tasmanian eucalyptus examined constitutes a limited subset of the largest plant genera globally, *Eucalyptus* serves as an invaluable system in investigating the evolution of traits underlying species rarity. This is due to the presence of numerous rare and endemic eucalypt species showcasing a diverse array of functional traits, life histories, and plant strategies. Through the utilization of this model system, these results suggest that species rarity can evolve, associated with an adaptive syndrome of traits such as was proposed by Rabinowitz (Rabinowitz, 1981) in 1981.

Under global change, rarity has and is expected to continue to increase on the landscape (Enquist et al., 2019). Identifying the suite of traits that may be associated with rarity and that have diverged phylogenetically is a significant frontier that is critical for predicting species persistence on the landscape with increasing fragmentation. Here we focused on biomass production as it has been linked to the evolution of range size (Stahl et al., 2014) and endemism (Gorman et al., 2014). However, rarity is likely influenced by a multitude of genetically based traits. For example, Gorman et al. (Gorman et al., 2014) showed that endemic species with restricted ranges were less productive and had tougher leaves with smaller specific leaf area. Gorman et al. (2014) also found that insect herbivory was lower, and the composition of insect communities were different on endemics relative to nonendemics. Consequently, the lower biomass of rare plant species may reflect trade‐offs with other functional traits and ecological processes, such as increased morphological complexity (Gurevich & Hadany, 2021), enhanced pollinator dynamics (Benadi & Gegear, 2018), and reduced insect herbivory (Bürli et al., 2023; Gorman et al., 2014).

Resource allocation (i.e., the proportion of resources given to above versus belowground biomass) is particularly important for light, water, and nutrient acquisition in resource limited, competitive environments (Umaña et al., 2021). Interestingly, patterns in above versus belowground biomass allocation were dependent on the range size (large versus small) distinction characterizing eucalypts; species with large range sizes allocated proportionally more resources belowground than aboveground. As demonstrated in Kleyer et al. (2019), biomass and biomass partitioning are critical drivers of trade‐offs between plant traits and strategies. Specifically, plant species that become larger in response to a range of abiotic and biotic factors must allocate more resources belowground for anchorage and storage rather than resource foraging (Kleyer et al., 2019). Our results demonstrate that species varying on other axes of rarity, such as habitat specificity or local population size, but characterized by large range sizes may allocate more resources to belowground biomass for storage and maintenance of these large ranges. In contrast, by investing more resources in aboveground biomass, rare plant species with small ranges may enhance their ability to compete and survive in light‐limited environments (Sterck et al., 2011), despite overall smaller size and reduced allocation to belowground structures. The increased ability of rare species to allocate resources to the aboveground biomass, rather than belowground structures as demonstrated in common species, may be facilitated by unique, microbially mediated plant–soil feedbacks (Maron et al., 2016) or may be indicative of a competitive response in mixture (Zhang et al., 2022). In contrast, patterns in biomass and resource allocation demonstrated by rare species may reflect a larger functional strategy rather than simply an ecological status. However, these results demonstrate how the selection for performance traits, such as above‐ and belowground biomass, related to rarity, could also have strong effects on plant community dynamics, plant–plant interaction outcomes, and ecosystem function.

Identifying the environmental gradients that may drive the evolution of rarity and traits important for surviving when rare is a novel ecological and evolutionary frontier. We found that rare species with low biomass occupied smaller ranges and more specific habitat types than related high biomass common species. The repeated convergent evolution of biomass across Tasmanian eucalypts suggests that ecological pressures and environmental constraints have shaped the adaptive strategies of species varying in rarity, leading to divergent optimizations of biomass within respective range sizes and habitats. Moreover, this convergent pattern provides a mechanism mediating the persistence of rare plant species on the landscape. Through the prioritization of aboveground biomass and maintenance of lower total biomass, rare species may enhance their persistence through effective niche differentiation and increased access to limited resources in shared habitats (Mi et al., 2012). Interestingly, traits associated with rare plant species, such as lower levels of biomass, decreased wood density, seed mass, and maximum height, are correlated with environmental variables that determine and constrain a species range (Stahl et al., 2014), suggesting that climate may be one such factor driving the evolution of rarity. For example, climate drives patterns of habitat loss, disturbance, and resource availability, which can act to shape the genetic selection and evolutionary trajectory of traits. These traits, under selection, play a large role in shaping the spatial and temporal distributions of species (i.e. less productive Tasmanian eucalypts have smaller ranges and more specific habitats). As rarity is prevalently measured by the range size, habitat specificity, and local population size of a species, trait‐based changes in geographic distribution result in the rarity or commonness of a species. Furthermore, stochastic processes may affect rare species at a greater rate because of these traits, therefore creating eco‐evolutionary feedback determining if and how a species is rare on the landscape. Environmental factors may also cause closely related species inhabiting similar niche space to diversify traits as a mechanism of escape from competitive exclusion or limiting resources (Lloyd et al., 2002; Zaneveld & Thurber, 2014). Other studies have shown that rates of herbivory, attack by soil pathogens, and variation in plant–soil feedbacks can differ in rare versus common plants (Gorman et al., 2014; Levine et al., 2010; Maron et al., 2016), any one of which can drive evolutionary trait divergence within and convergence among subgenera (Agrawal et al., 2012; Schweitzer et al., 2014) with subsequent effects on species rarity.

### 4.1 Implications

Species rarity is common and predicted to become more common (Enquist et al., 2019). If reduced biomass production is a fundamental trait associated with plant rarity, as we have shown here with *Eucalyptus*, then there are likely to be significant impacts on the associated biodiversity and ecosystem function critical for climate change mitigation (Lal, 2004; Shahzad et al., 2022). Although soil composition and edaphic properties play roles in carbon storage ability, the above‐ and belowground biomass of plants act as the largest potential pools of carbon storage and sequestration (de Oliveira et al., 2019; Meena et al., 2019). Although the dynamics between traits, patterns of geographic occurrence, and systemic rarity need to be examined across a wide variety of plant species (especially herbaceous genera), as rare and common species distributions and associated biomass production shift in favor of rare plant species under variable global change factors, previously captured carbon may be lost as future sequestration abilities and patterns change (Gayathri et al., 2021). These results suggest that understanding patterns of rarity on the landscape are no longer a population‐level phenomenon representing a conservation priority, but rather an interplay of evolutionary and ecological factors that must be considered in greater depth.

## AUTHOR CONTRIBUTIONS

**Alivia G. Nytko:** Formal analysis (lead); investigation (lead); visualization (lead); writing – original draft (lead); writing – review and editing (lead). **John K. Senior:** Conceptualization (equal); data curation (lead); methodology (equal); project administration (lead); writing – review and editing (supporting). **Rachel C. Wooliver:** Resources (equal); software (equal); writing – review and editing (supporting). **Julianne O'Reilly‐Wapstra:** Conceptualization (equal); methodology (equal); writing – review and editing (supporting). **Jennifer A. Schweitzer:** Conceptualization (equal); methodology (equal); writing – review and editing (supporting). **Joseph K. Bailey:** Conceptualization (equal); investigation (equal); methodology (equal); supervision (lead); writing – original draft (supporting); writing – review and editing (equal).

## CONFLICT OF INTEREST STATEMENT

The authors declare that there are no conflicts of interest.

## Supporting information


AppendixS1

## Data Availability

The data that support the findings of this study are available in figshare (https://doi.org/10.6084/m9.figshare.24128733.v2).
