# Supplementary material for: An evolutionary case for plant rarity: Eucalyptus as a model system
Source: Ecol Evol. 2024 Jun 6;14(6):e11440. doi: 10.1002/ece3.11440 (PMC11156952; doi:10.1002/ece3.11440)
Supplement: Supplementary file 1 — AppendixS1 [file ECE3-14-e11440-s001.docx]

Supporting Information File 1

S1_File

Includes: S1-S4 Tables and S1-S3 Figures

**S1 Table. Categorization of 25 species of Tasmanian *Eucalyptus* by rarity level.**

| **Species** | **Life Form** | | **Rarity** |
| --- | --- | --- | --- |
| *E. perriniana* | Shrub | 1 | |
| *E. radiata* | Tree | 1 | |
| *E. risdonii* | Shrub | 1 | |
| *E. urnigera* | Mallee | 2 | |
| *E. barberi* | Tree | 3 | |
| *E. cordata* | Tree | 3 | |
| *E. johnstonii* | Tree | 3 | |
| *E. pulchella* | Tree | 3 | |
| *E. sieberi* | Tree | 3 | |
| *E. brookeriana* | Tree | 4 | |
| *E. rubida* | Tree | 4 | |
| *E. subcrenulata* | Tree | 4 | |
| *E. tenuiramis* | Tree | 5 | |
| *E. vernicosa* | Mallee | 5 | |
| *E. dalrympleana* | Mallee | 6 | |
| *E. gunnii* | Tree | 6 | |
| *E. nitida* | Tree | 6 | |
| *E. rodwayi* | Tree | 6 | |
| *E. globulus* | Tree | 7 | |
| *E. regnans* | Tree | 7 | |
| *E. amygdalina* | Tree | Common | |
| *E. delegatensis* | Tree | Common | |
| *E. obliqua* | Tree | Common | |
| *E. ovata* | Tree | Common | |
| *E. viminalis* | Tree | Common | |

**S2 Table. Preliminary linear mixed model (LMM) examining the fixed singular and interacting effects of CO_2_ addition, Nitrogen (N) enrichment, rarity level, and life form on total biomass across all 25 species of Tasmanian *Eucalyptus*.** Alpha = 0.05.

| **Response** | **Sum sq** | **DF** | **F value** | **P value** |
| --- | --- | --- | --- | --- |
| CO_2_ Addition | 4.966 | 1 | 25.329 | 6.304e-07* |
| Nitrogen (N) Enrichment | 6.465 | 1 | 32.970 | 1.452e-08* |
| Rarity | 14.334 | 7 | 10.444 | 1.900e-12* |
| Life Form | 0.050 | 2 | 0.129 | 0.879 |
| CO_2_ Addition x N Enrichment | 4.241 | 1 | 21.627 | 4.031e-06* |
| CO_2_ Addition x Rarity | 1.532 | 7 | 1.116 | 0.351 |
| N Enrichment x Rarity | 2.757 | 7 | 2.009 | 0.052 |
| CO_2_ Addition x N Enrichment x Rarity | 0.587 | 7 | 0.428 | 0.885 |

**S3 Table. Summary statistics for PGLS models (biomass ~ rarity + life form).** Alpha = 0.05.

| **Response** | **Covariate** | **Estimate** | **Std. Error** | **t value** | **P value** |
| --- | --- | --- | --- | --- | --- |
| Total biomass | Intercept | -1.054 | 2.340 | -0.451 | 0.659 |
|  | Rarity Level 2 | 0.601 | 0.399 | 1.506 | 0.153 |
|  | Rarity Level 3 | 0.557 | 0.235 | 2.365 | 0.032* |
|  | Rarity Level 4 | 0.563 | 0.259 | 2.175 | 0.046* |
|  | Rarity Level 5 | 0.384 | 0.265 | 1.451 | 0.167 |
|  | Rarity Level 6 | 0.471 | 0.236 | 1.994 | 0.065 |
|  | Rarity Level 7 | 0.594 | 0.317 | 1.870 | 0.081 |
|  | Common | 0.630 | 0.210 | 2.998 | 0.009* |
|  | Life Form Shrub | 0.036 | 0.363 | 0.099 | 0.922 |
|  | Life Form: Tree | 0.105 | 0.263 | 0.446 | 0.662 |
| Aboveground biomass | Intercept | -1.216 | 2.201 | -0.552 | 0.589 |
|  | Rarity Level 2 | 0.590 | 0.375 | 1.572 | 0.137 |
|  | Rarity Level 3 | 0.543 | 0.221 | 2.454 | 0.027* |
|  | Rarity Level 4 | 0.516 | 0.243 | 2.119 | 0.051 |
|  | Rarity Level 5 | 0.365 | 0.249 | 1.467 | 0.163 |
|  | Rarity Level 6 | 0.468 | 0.222 | 2.107 | 0.052 |
|  | Rarity Level 7 | 0.574 | 0.299 | 1.924 | 0.074 |
|  | Common | 0.601 | 0.198 | 3.040 | 0.008* |
|  | Life Form: Shrub | 0.041 | 0.341 | 0.119 | 0.907 |
|  | Life Form: Tree | 0.110 | 0.222 | 0.494 | 0.628 |
| Belowground biomass | Intercept | -2.979 | -2.979 | -0.964 | 0.350 |
|  | Rarity Level 2 | 0.675 | 0.526 | 1.283 | 0.219 |
|  | Rarity Level 3 | 0.624 | 0.311 | 2.010 | 0.063 |
|  | Rarity Level 4 | 0.780 | 0.342 | 2.282 | 0.037* |
|  | Rarity Level 5 | 0.499 | 0.349 | 1.430 | 0.173 |
|  | Rarity Level 6 | 0.511 | 0.312 | 1.639 | 0.122 |
|  | Rarity Level 7 | 0.691 | 0.419 | 1.649 | 0.120 |
|  | Common | 0.791 | 0.277 | 2.853 | 0.012* |
|  | Life Form: Shrub | 0.023 | 0.479 | 0.048 | 0.963 |
|  | Life Form: Tree | 0.099 | 0.311 | 0.317 | 0.756 |

**S4 Table. Summary statistics for PGLS models examining the singular and interactive effects of total biomass and subgenera on species’ range size, habitat specificity, and population aggregation.** Alpha = 0.05.

| **Response** | **Covariate** | **Estimate** | **Std. Error** | | **t value** | **P value** |
| --- | --- | --- | --- | --- | --- | --- |
| Range Size | Intercept | 492.63 | 1677.52 | 0.294 | | 0.772 |
|  | Total biomass | 434.17 | 121.18 | 3.583 | | 0.002* |
|  | Subgenera: Symphyomyrtus | -329.64 | 2368.18 | -0.139 | | 0.891 |
|  | Total biomass x Subgenera: Symphyomyrtus | -315.91 | 168.84 | -1.871 | | 0.075 |
| Habitat Specificity | Intercept | 1.223 | 3.469 | 0.353 | | 0.728 |
|  | Total biomass | 0.858 | 0.251 | 3.426 | | 0.003* |
|  | Subgenera: Symphyomyrtus | -0.631 | 4.897 | -0.129 | | 0.899 |
|  | Total biomass x Subgenera: Symphyomyrtus | -0.643 | 0.349 | -1.842 | | 0.080 |
| Population Aggregation | Intercept | 50.840 | 181.158 | 0.281 | | 0.782 |
|  | Total biomass | -25.854 | 13.087 | -1.976 | | 0.061 |
|  | Subgenera: Symphyomyrtus | -5.078 | 255.744 | -0.020 | | 0.984 |
|  | Total biomass x Subgenera: Symphyomyrtus | 34.260 | 18.283 | 1.879 | | 0.074 |

**S1 Fig. Classification of rank ordered rarity levels using geographic range, habitat specificity, and local population size [2].**

**
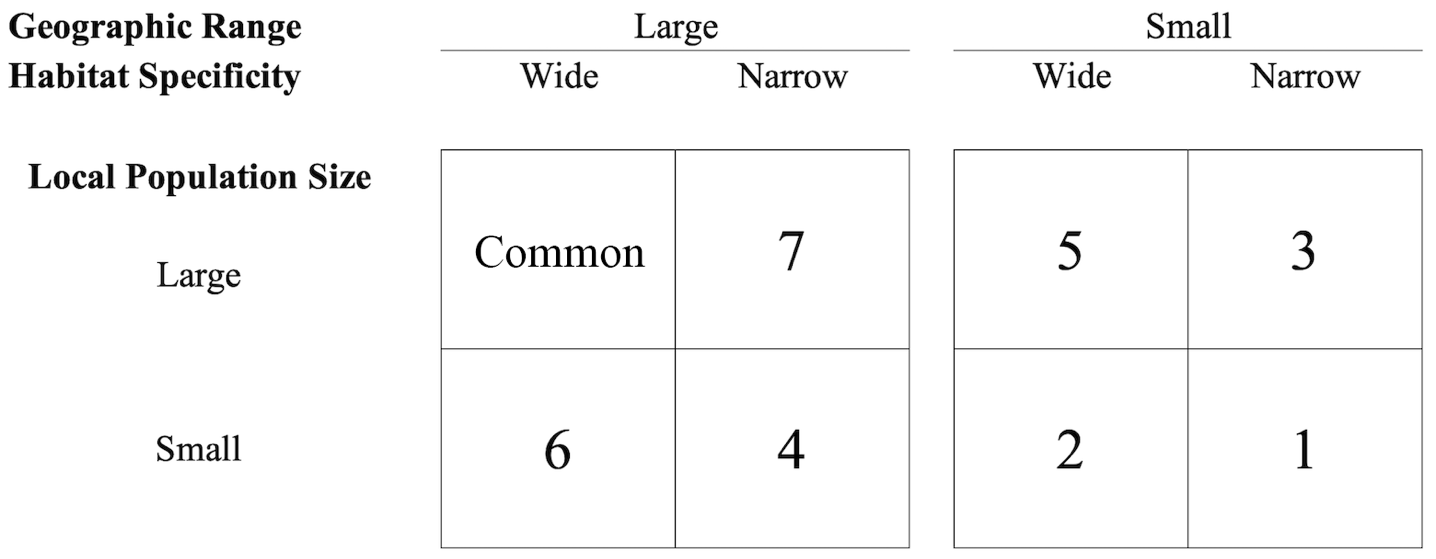
**

**S2 Fig. Full factorial experimental design consisting of species mixtures varying in rarity under varying treatments of Nitrogen (N) fertilization and CO2 enrichment.** All possible rarity combinations were represented among four types of N fertilization and CO_2_ enrichment. Treatments such as high N/high CO2, high N/low CO2, low N/high CO2, and low N/low CO2 were distributed equally among mixtures varying in rarity.

**
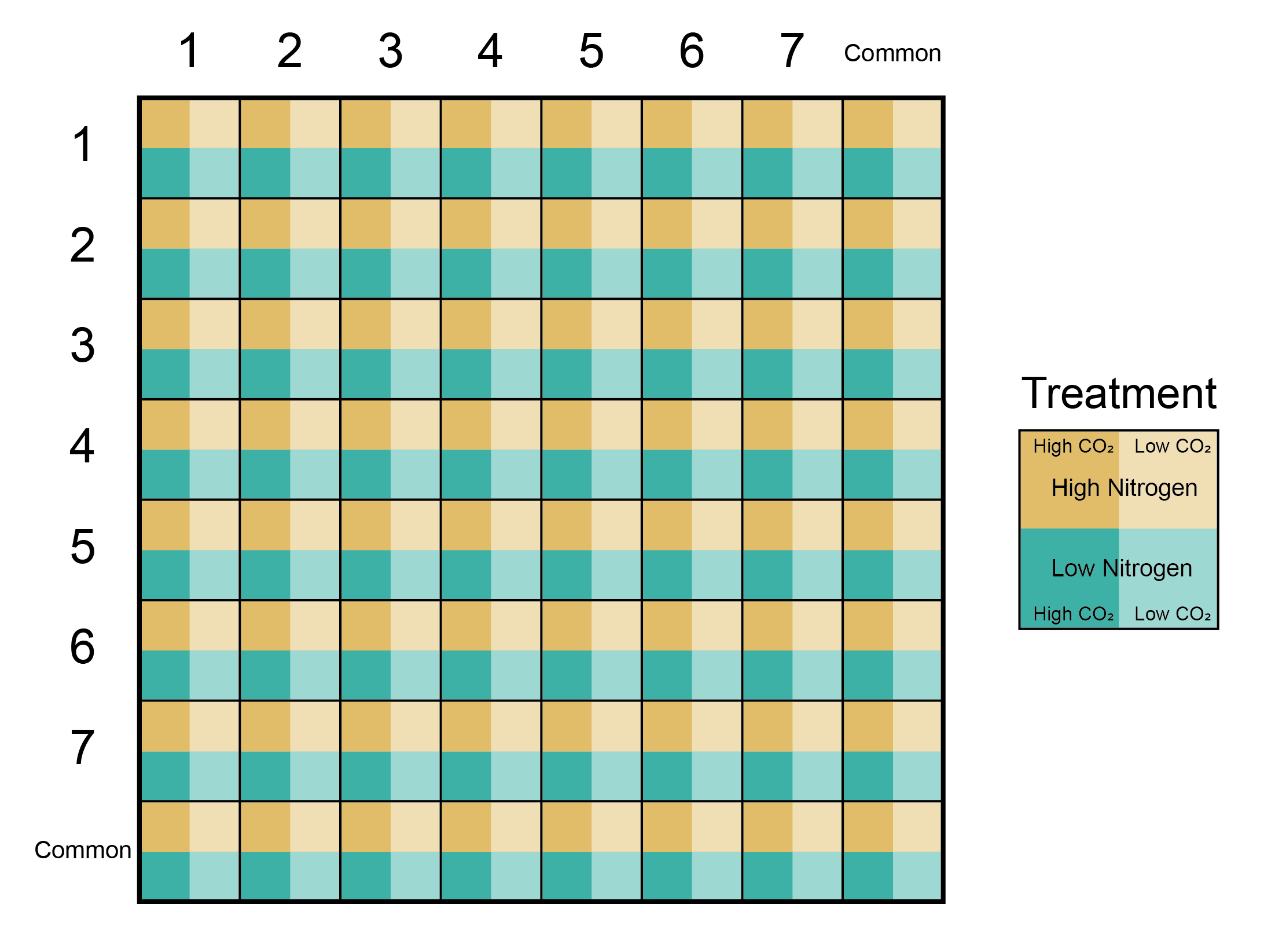
**

**S3 Fig. Relationship between seedling biomass and mean adult height of Tasmanian *Eucalyptus* across two subgenera.** The relationship between total seedling biomass in the common garden and mean adult height in natural populations is positively correlated across both subgenera (r = 0.28, p-value = 1.602e-13) [59].

**
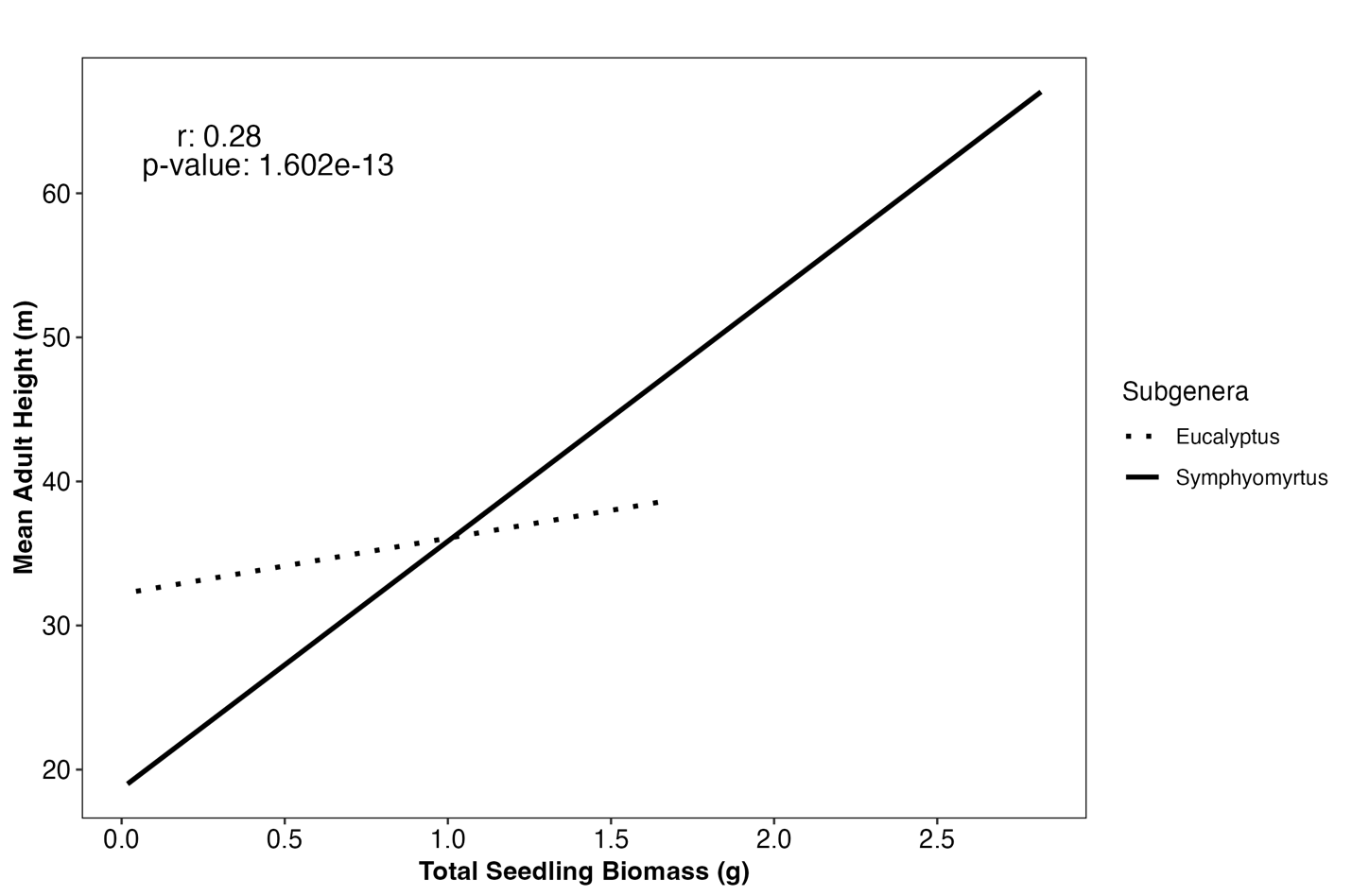
**
